# Partial small intestinal resection for successful surgical management of refractory protein-losing gastroenteropathy in systemic lupus erythematosus: A case report and literature review: Retraction

**DOI:** 10.1097/MD.0000000000015400

**Published:** 2019-04-19

In the article “Partial small intestinal resection for successful surgical management of refractory protein-losing gastroenteropathy in systemic lupus erythematosus: A case report and literature review”,^[1]^ which appeared in Volume 97, Issue 30 of *Medicine*, the authors stated that they had patient consent and parental consent. However, the author group was unable to provide this consent when proof was requested.

## References

[1] Iwasaki K Morimoto M Ota G Partial small intestinal resection for successful surgical management of refractory protein-losing gastroenteropathy in systemic lupus erythematosus: A case report and literature review. *Medicine*. 2018 97:e11357. 30045258 10.1097/MD.0000000000011357 PMC6078739

